# Narrating the psychoneuroimmunomodulatory properties of serotonin 5-HT_2A_ receptor psychedelics from a transdiagnostic perspective

**DOI:** 10.1017/neu.2025.10030

**Published:** 2025-07-25

**Authors:** Guillaume Thuery, Christopher Sheridan, Patricia Iusan, Gurjot Brar, Kathryn Ledden, Aoife Freyne, John R. Kelly, Andrew Harkin

**Affiliations:** 1 Psychedelic Research Group, Tallaght University Hospital and Trinity College Dublin, Dublin, Ireland; 2 Department of Psychiatry, School of Medicine, Trinity College Dublin, Dublin, Ireland; 3 Neuropsychopharmacology Research Group, Trinity College Institute of Neuroscience, Dublin, Ireland; 4 School of Pharmacy and Pharmaceutical Sciences, Trinity College Dublin, Dublin, Ireland

**Keywords:** serotonin, 5-HT2A Receptor, psychedelics, psychoneuroimmunology, psychiatric disorders

## Abstract

**Objective:**

By synthesising findings from both clinical and preclinical research, this review aims to provide an understanding of the interplay between 5-HT2A receptor psychedelics and the immune system and considers how their immunomodulatory effects associate with neuronal and behavioural changes.

**Methods:**

A PubMed literature search covering the past 30 years was conducted using keywords such as ‘5-HT2A receptor’, ‘psychedelics’, ‘immune system’, and ‘HPA axis’. Studies were included if they addressed the effects of 5-HT2AR psychedelics on immune function, neuroimmune interactions, or HPA axis involvement. This narrative review synthesises evidence highlighting the bi-directional effects of 5-HT2AR psychedelics between the immune and nervous systems, identified through this search process.

**Results:**

Preclinical and clinical studies report that 5-HT2AR psychedelics have some direct immunomodulatory properties with downregulation of gene regulators like NF-κB, and reduced cytokine expression such as TNF-α, IL-6, and IL-1β at a central and peripheral level, accompanied by modulation of corticotrophin releasing hormone (CRH), adrenocorticotrophic hormone (ACTH), and cortisol. Direct immunomodulatory effects are mediated by pathways involving serotonin receptors, the Sigma-1 receptor, and the TrkB receptor. Immunomodulation is further mediated indirectly via the HPA axis.

**Conclusion:**

Further studies will determine the molecular and cellular mechanisms underlying these immunomodulatory effects. There is growing interest in the potential of 5-HT2AR psychedelics for treating a range of mental health and brain disorders. In keeping with their immunomodulatory actions, the likely modulation of brain glia and glial-neuronal interaction remains to be determined, representing a promising direction of further research on the therapeutic potential of 5-HT2AR psychedelics.

Summations

- Preclinical *in vitro* and *in vivo* studies indicate 5-HT_2A_R psychedelics have immunomodulatory properties.
- Immunomodulatory effects are mediated by pathways involving serotonin receptors, the Sigma-1 receptor (SIG-1R), and the TrkB receptor.
- Further investigation is warranted to understand their influence on glial cells, glial-neuronal interactions, and neuroinflammatory processes.

Considerations

- Investigation with consistent experimental protocols is warranted to elucidate underlying mechanisms.
- Additional well-designed clinical investigations will be crucial to definitively assess the impact of psychedelics on the human immune system.
- Combined with existing post-mortem imaging techniques in animals, imaging modalities like MRI scans allow researchers to directly correlate functional connectivity and other MRI measures with neuroplasticity, glial activation, neuroinflammation, and neuronal cell death to further elucidate the immunoregulatory properties of psychedelics with impacts on the nervous system.

## Introduction

Research on serotonin 5-hydroxytryptamine (5-HT)-2A receptor (5-HT_2A_R) psychedelic drugs, namely lysergic acid diethylamide (LSD), psilocybin, or *N*,*N*-dimethyltryptamine (*N*,*N*-DMT), has experienced a resurgence in recent years. This has been focused on their therapeutic effects for the treatment of various mental disorders (Griffiths *et al*., 2016; Carhart-Harris *et al*., 2017, 2018, 2021; Mertens *et al*., 2020; Doss *et al*., 2021 Raison *et al*., 2023; Goodwin *et al*., 2023a, c), with hundreds of registered clinical trials underway.

Researchers and biotechnology companies are exploring how psychedelic drugs may be used in a clinical setting as an additional treatment modality to address the growing mental health challenges that have become more pronounced in the wake of the COVID-19 pandemic (Akil *et al*., 2010; Nissen *et al*., 2020; Jones *et al*., 2021; Oliveira *et al*., 2022; Moncrieff *et al*., 2023).

Most clinical and preclinical studies are focusing on how these drugs can induce changes in neurocircuitry and neuroplasticity in the short- and long-term (Preller *et al*., 2018; Shao *et al*., 2021, 2024; Raval *et al*., 2021; Grieco *et al*., 2022; Inserra *et al*., 2023; Funk *et al*., 2024), with what seems to be promising therapeutic effects in the context of mental health (Castren and Antila, 2017; Olson, 2018; Grieco *et al*., 2022).

Evidence is also emerging to indicate that these drugs affect the immune system and peripheral areas including the gut and vascular systems. The immune system is a complex network of different organs, cell types, and chemical mediators which may be directly or indirectly modulated by psychedelics. The numerous interactions between organs, cells, and soluble mediators have been extensively reviewed elsewhere (Carpenter and O’Neill, 2024; Delves *et al*., 2017; Marshall *et al*., 2018).

Gut-immune-brain interactions further complicate our understanding of how psychedelics cause psychological effects. Although research on psychedelics for mental health disorders is advancing rapidly, exploring their impact on the immune and neuroendocrine systems in humans is also showing promise. Studies to date of 5-HT_2A_R psychedelics exploring their impact on bi-directional links between the immune and central nervous system (CNS), and their psychological effects have yielded mixed results, prompting a review of their psychoneuroimmunological properties.

A literature search was performed using PubMed, focusing on articles published within the last 30 years. Relevant keywords and phrases included combinations such as ‘5-HT2A receptor’, ‘psychedelics’, ‘immune system’, ‘HPA axis’, and related terms. The search strategy aimed to capture studies investigating both the acute and long-term effects of 5-HT_2A_R psychedelics on immune function, as well as their indirect effects mediated through the nervous system and the hypothalamic-pituitary-adrenal (HPA) axis. Articles were initially screened by title and abstract for relevance to the review’s focus. Full-text articles were subsequently reviewed if they met the inclusion criteria, which required that studies addressed the effects of 5-HT_2A_R psychedelics on immune parameters, neuroimmune interactions, or HPA axis involvement. A summary of the *in vitro* and *in vivo* studies having measured the immunomodulatory properties of 5-HT psychedelics can be found in Tables 1 and 2 respectively.

### Peripheral immune system signalling to the brain

The relationship between the peripheral immune system, the CNS, and brain resident glial cells is an ongoing area of research. Peripheral immune cells at the choroid plexus (CP), blood brain barrier (BBB), and meninges participate in the transport of antigens from the CNS to lymph nodes via the cerebrospinal fluid (CSF) (Rustenhoven and Kipnis, 2022). Activation of the immune system in the periphery signals in turn to the CNS. Disruption of the CP and BBB may allow immune cells to enter the brain, while immune signalling molecules like cytokines and chemotactic factors cross into the CNS via cerebrovascular endothelial transporters (Millett *et al*., 2022). The infiltration of immune system mediators from the periphery into the CNS triggers the mobilisation of glial cells in the brain.

Viral infections, for instance the severe acute respiratory syndrome coronavirus 2 (SARS-CoV-2), may trigger sustained peripheral inflammatory responses with production of interleukin (IL)-6, tumour necrosis factor (TNF)-α, adipokines, chemo-attractants, and reactive oxygen species (ROS) which can disrupt BBB permeability (Brundin *et al*., 2020). These effects are particularly relevant given the prevalence of long-COVID, which often involves persistent psychological and neurological symptoms after infection (Wong *et al*., 2023; Greene *et al*., 2024). Furthermore, gut microbiota disruption and life stress, signal to the brain via the immune system which impact on behaviour and symptoms mediated through the CNS (Zhang *et al*., 2023b).

Beyond innate immune mechanisms, disruption of BBB permeability can also allow circulating autoantibodies from the adaptive immune system to enter the brain parenchyma. Under normal conditions, the BBB prevents brain-reactive antibodies from causing brain pathology. However, under pathological conditions, these antibodies can penetrate the CNS and bind to various neuronal and non-neuronal targets (Diamond *et al*., 2013). These autoantibodies can modulate microglial activation and function, directly affect neural signalling by interacting with neurotransmitter receptors, and contribute to neuroinflammation and tissue damage. For instance, in systemic lupus erythematosus, autoantibodies that cross-react with neuronal N-methyl-D-aspartate (NMDA) receptors can lead to neurocognitive dysfunction, while in neuromyelitis optica, antibodies targeting astrocytic aquaporin-4 cause astrocyte damage (Mader *et al*., 2017). Recent perspectives have transformed our understanding of brain immunity from viewing the brain as isolated to recognising the complex bi-directional communication between the CNS and the immune system through various interfaces and compartments at the brain’s borders (Castellani *et al*., 2023), with important implications for neurological and psychiatric disorders.

### Glial compartments

Astrocytes, microglia, and oligodendrocytes support neuronal function in the CNS and facilitate the connection between the CNS and the immune system. Astrocytes support neuronal function and maintain BBB integrity but can also contribute to neuroinflammation through cytokine release (Cekanaviciute and Buckwalter, 2016). Microglia are the primary immune effector cells within the CNS and play a pivotal role in response to the presence of pathogens. Activated microglia can adopt a variety of phenotypes, displaying either predominantly pro-inflammatory (M1) or anti-inflammatory (M2) characteristics. However, their activation states are not strictly limited to these two categories and are more complex than a simple M1/M2 distinction (Gao *et al*., 2023; Wang *et al*., 2023). Microglia release a variety of factors such as cytokines which can target neuronal cells (Ransohoff and Brown, 2012) and influence synaptic plasticity and other CNS functions (Werneburg *et al*., 2017; Cornell *et al*., 2022). The relationship between microglia and astrocytes is important in the immune response of the brain, as they communicate bidirectionally (Norden *et al*., 2014; Bhusal *et al*., 2023). Glial cells play a role in maintaining CNS homeostasis through their gatekeeper function at the BBB, the expression of various factors can impact the function of this barrier, which is important in the context of inflammatory insults (Alvarez *et al*., 2013). Glial cells can also signal to BBB endothelial cells to increase monocyte trafficking into the CNS (Weber *et al*., 2017).

### CNS signalling to the immune system

The bi-directional relationship between the CNS and the immune system has been well reviewed (Maier, 2003; Wrona, 2006; Kamimura *et al*., 2020; Gentile *et al*., 2021). Efferent sympathetic and parasympathetic innervation of all lymphoid organs allows the CNS to release noradrenaline or acetylcholine and influence immune functions such as thymocyte maturation, T-cell development, and cytokine release (Kavelaars, 2002; Leposavic *et al*., 2011; Sundman and Olofsson, 2014; Carnevale *et al*., 2014; Dubeykovskaya *et al*., 2016; Chen *et al*., 2021; Francelin *et al*., 2021).

In addition to these sympathetic and vagal influences, the hypothalamic-pituitary-adrenal (HPA) axis introduces an indirect communication pathway between the CNS and the immune system. Acute and chronic environmental stress activates the HPA axis, leading to the release of glucocorticoids into the blood (Bellavance and Rivest, 2014). Cortisol is a known immunosuppressor and leads to anti-inflammatory effects via mitogen-activated kinase (MAPK1)-dependent and nuclear factor kappa B (NF-κB)-dependent pathways (Coutinho and Chapman, 2011; Zefferino *et al*., 2021). However, chronic stress can dysregulate the HPA axis, cause glucocorticoid resistance, and decreased expression of glucocorticoid receptors, contributing to immune dysregulation (Cohen *et al*., 2012; Silverman and Sternberg, 2012; Lam *et al*., 2022).

## A focus on serotonin and 5-HT_2A_R in neuroimmunomodulation

There is a total of fourteen known 5-HT receptors classified into seven families (5-HT_1_R–5-HT_7_R) based on their structural and functional properties. These have different downstream cellular effects, leading to increased or decreased cellular levels of cyclic adenosine monophosphate (cAMP), inositol triphosphate (IP_3_), and diacylglycerol (DAG), producing inhibitory or excitatory neuromodulatory responses (Frazer and Hensler, 1999).

### 5-HT_2A_R agonists

**Figure 1.**
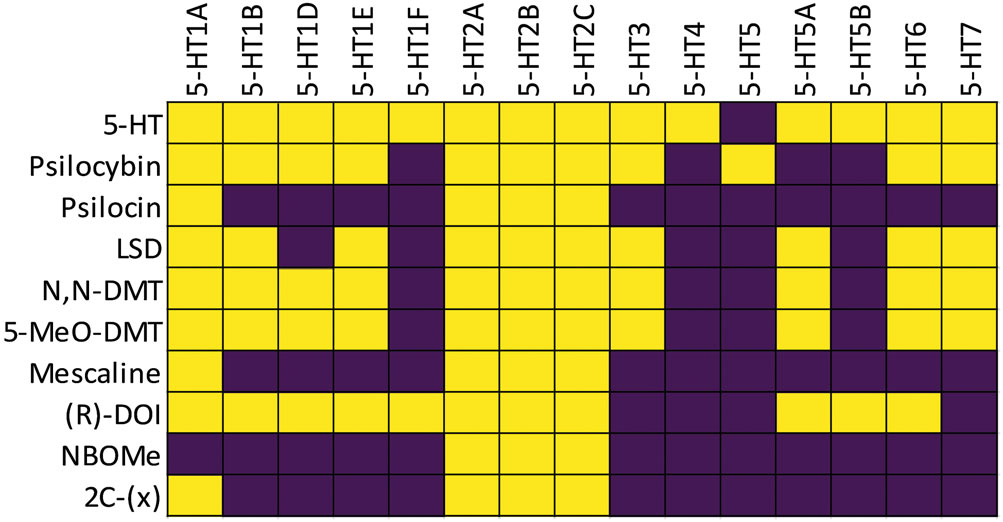
**Heat map visualisation of serotonergic receptor binding profiles.** Yellow cells with crosses indicate known binding activity of ligands (rows) to specific 5-HT receptors (columns), while dark purple cells indicate no reported binding. Data compiled from: Nichols (2004); Kitson (2007); Keiser *et al*. (2009); Besnard *et al*. (2012); Rickli *et al*. (2015); Wsol (2023); Hatzipantelis and Olson (2024); Ippolito *et al*. (2024). 2C-(x) refers to the family of 2,5-dimethoxy-phenethylamine analogues. Note that binding affinity varies based on pharmacological method, cell type, and experimental conditions. The psychoactive drug screening programme (PDSP) has been a primary source for standardised binding data (Ki values) for many of these compounds, as reviewed in Alexander *et al*. (2024); Hatzipantelis and Olson (2024).

Psychedelics have a wide range of binding profiles with the 5-HT receptor family (Ray, 2010) (Figure 1). The 5-HT_2A_R is of particular interest in the field of psychedelic research as it is largely associated with the psychotropic effects of psychedelics (Nichols, 2004; Preller *et al*., 2018; Madsen *et al*., 2019; Shao *et al*., 2025). This receptor is widely distributed throughout the cortex, whereas moderate to low levels of expression can be found in the limbic system, including the amygdala and the hippocampus, which plays a crucial role in emotional processing, memory formation, and behavioural regulation (Saulin *et al*., 2012).

Psychedelics can induce different downstream effects after activation of the 5-HT_2A_R. Psychedelics display biased agonism, also known as functional selectivity, referring to the ability of different ligands to selectively stabilise distinct receptor conformations, leading to the activation of specific signalling pathways over others (Inserra *et al*., 2021).

In the context of 5-HT receptors, psychedelics can induce alternative 5-HT receptor conformations, leading to altered ligand affinity recruitment of intracellular effector proteins, including β-arrestins, which mediate receptor desensitisation, internalisation, and signalling pathways independent of G proteins. For example, LSD induces a receptor conformational change that will preferentially recruit β-arrestin. Interestingly, psychedelic-related effects induced by LSD appear to be mediated by β-arrestin (Rodriguiz *et al*., 2021) unlike the full 5-HT_2A_R agonist 2,5-dimethoxy-4-iodoamphetamine [(*R*)-DOI] (Schmid *et al*., 2008).

A recent meta-analysis found that there are no significant differences in selectivity between *N,N*-DMT, LSD, and psilocin, relative to the 5-HT_1A_R, but they reported that LSD induced a significantly higher formation of inositol phosphate (Shinozuka *et al*., 2024).

The 5-HT_2C_R has been shown to be involved in the head-twitch response (HTR), a commonly used measure for a hallucinogenic-like experience in rodents. It remains unclear exactly how this receptor contributes to this behavioural response as some studies have found that antagonists increase – and agonists attenuate – the HTR (Canal *et al*., 2013; Erkizia-Santamaria *et al*., 2022), whereas some studies have found that its activation can induce this behaviour (Custodio *et al*., 2023).

Psychedelics mediate their effects through 5-HT_2A_ receptors, to distinct signalling and functional outcomes that occur depending on the localisation of receptor activation (Olson, 2021). Extracellular 5-HT_2A_ activation by psychedelics like psilocin initiates Gq/11-PLC signalling, which exceeds 70% efficacy threshold to induce hallucinations and HTR, while sub-threshold agonists (e.g., lisuride) lack psychedelic effects (de Vos *et al*., 2021; Wallach *et al*., 2023). β-Arrestin2 recruitment via cell-surface receptors does not correlate with psychedelic potential or with the psychedelic experience and may instead promote receptor downregulation (Schmid and Bohn, 2010).

Lipophilic psychedelics (e.g., DMT) activate intracellular 5-HT2A pools, triggering neuroplasticity via transglutaminase 2 (TGM2)-mediated serotonylation of Rac1, which enhances dendritic arborisation and synaptogenesis (Ly *et al*., 2018; de la Fuente Revenga *et al*., 2021). This intracellular signalling may contribute to the sustained therapeutic effects observed in clinical trials for the treatment of depression, independent of hallucinogenic activity (Vargas *et al*., 2023). Emerging strategies aim to decouple neuroplasticity from psychedelic effects by targeting intracellular receptors, using specific β-arrestin modulators or designing Gq-sub-threshold agonists, offering a pathway for non-hallucinogenic therapeutics (Dunlap *et al*., 2020; Wallach *et al*., 2023).

β2-arrestin biased signalling modulates MAPK pathways such as ERK1/2, JNK, and p38, influencing immune cell activity through scaffold-mediated regulation. β-arrestins form complexes with ERK1/2, sequestering it in the cytosol and preventing nuclear translocation, altering transcription, and dampening pro-inflammatory cytokine production (Sharma and Parameswaran, 2015). In macrophages, β-arrestin2 inhibits TLR2/ERK1/2 signalling, reducing TNF-α expression (Fan, 2014). β-arrestin1 promotes CD4^+^ T-cell survival by upregulating Bcl2, while β-arrestin2 suppresses NK cell cytotoxicity by interacting with inhibitory receptors (Crepieux *et al*., 2017). β-arrestin2 also negatively regulates NF-κB by stabilising IκBα, limiting inflammatory cytokine release in sepsis models (Fan, 2014). β-arrestin appears to act in a modulatory fashion in immune regulation, balancing pro- and anti-inflammatory responses through modulation of MAPK activity and crosstalk with PRR signalling (Sharma and Parameswaran, 2015).

Certain immune cells, including monocytes, macrophages, dendritic cells, and T cells, express high levels of 5-HT_2A_ receptors making them functionally sensitive to co-ligation by psychedelics that target 5-HT receptors and activate β-arrestin signalling through crosstalk with PRR pathways such as TLRs, and can influence key signalling cascades like NF-κB and MAPK. β-arrestin-biased agonists at 5-HT_2A_Rs can further fine-tune these effects, potentially reducing pro-inflammatory responses (Nau *et al*., 2013; Szabo, 2015; Flanagan and Nichols, 2018).

### 5-HT_2A_R and regulation of brain glia

Brain glia, and specifically microglia, have been shown to express various forms of 5-HT receptors including the 5-HT_2A_R (Krabbe *et al*., 2012; Glebov *et al*., 2015) and 5-HT_2B_R influencing microglial development (Kolodziejczak *et al*., 2015; Turkin *et al*., 2021). Although the exact functions of these remain unclear, studies to date suggest that activation of these 5-HT_2A_R promotes the release of microglia-derived vesicles known as exosomes containing a variety of proteins and ribonucleic acids (RNA) (Glebov *et al*., 2015). 5-HT_2A_R influences the dynamic extensions of microglia which play an important role in surveillance and maintenance functions within the CNS (Krabbe *et al*., 2012). Microglia may play a role in the neuropharmacological and therapeutic effects of various drugs, including dissociative NMDA receptor psychedelics like ketamine and 5-HT_2A_R psychedelics (VanderZwaag *et al*., 2023).

Astrocytes express multiple forms of 5-HT receptors, including the 5-HT_2A_R (Hagberg *et al*., 1998; Hirst *et al*., 1998; Cohen *et al*., 1999; Maxishima *et al*., 2001; Kong *et al*., 2002; Verkhratsky *et al*., 2021). Although more research is required, 5-HT_2A_R seems to play a role in 5-HT-driven astrocytic calcium signalling with a possible role in synaptic plasticity (Jalonen *et al*., 1997; Hagberg *et al*., 1998; Gonzalez-Arias *et al*., 2023). An immunocytochemical study found increased 5-HT_2A_R expression in astrocytes within the prefrontal cortex of Alzheimer’s disease patients and the caudate nucleus of Huntington’s disease patients (Wu *et al*., 1999). Astrocytes also express the serotonin transporter (SERT) (Fitzgerald *et al*., 1990; Bel *et al*., 1997), enabling them to regulate the extracellular availability of 5-HT (Edmondson *et al*., 2007).

Oligodendrocytes form the myelin sheath around neuronal axons and support neuronal plasticity (Jang *et al*., 2019). Both *in vitro* and *in vivo* studies demonstrate that these cells express the 5-HT_2A_R, with elevated serotonin levels altering myelination via this receptor (Simpson *et al*., 2011; Fan *et al*., 2015). Psilocybin elicits an increase in cellular activation indicated by expression of the immediate early gene c-Fos in neurons and in oligodendrocytes (Funk *et al*., 2024). This increase was found in 10–20% of neurons and 25% of oligodendrocytes in the medial prefrontal cortex (mPFC), basolateral amygdala, and the dorsal raphe nucleus of male rats.

### Effect of 5-HT on the immune system

In the periphery, most 5-HT production occurs in enterochromaffin cells located in the gut. Gut-derived 5-HT modulates gastrointestinal functions and immune cells in or near the gut epithelium. It can also enter the bloodstream, where platelets absorb it via SERT (Cloutier *et al*., 2018).

**Figure 2.**
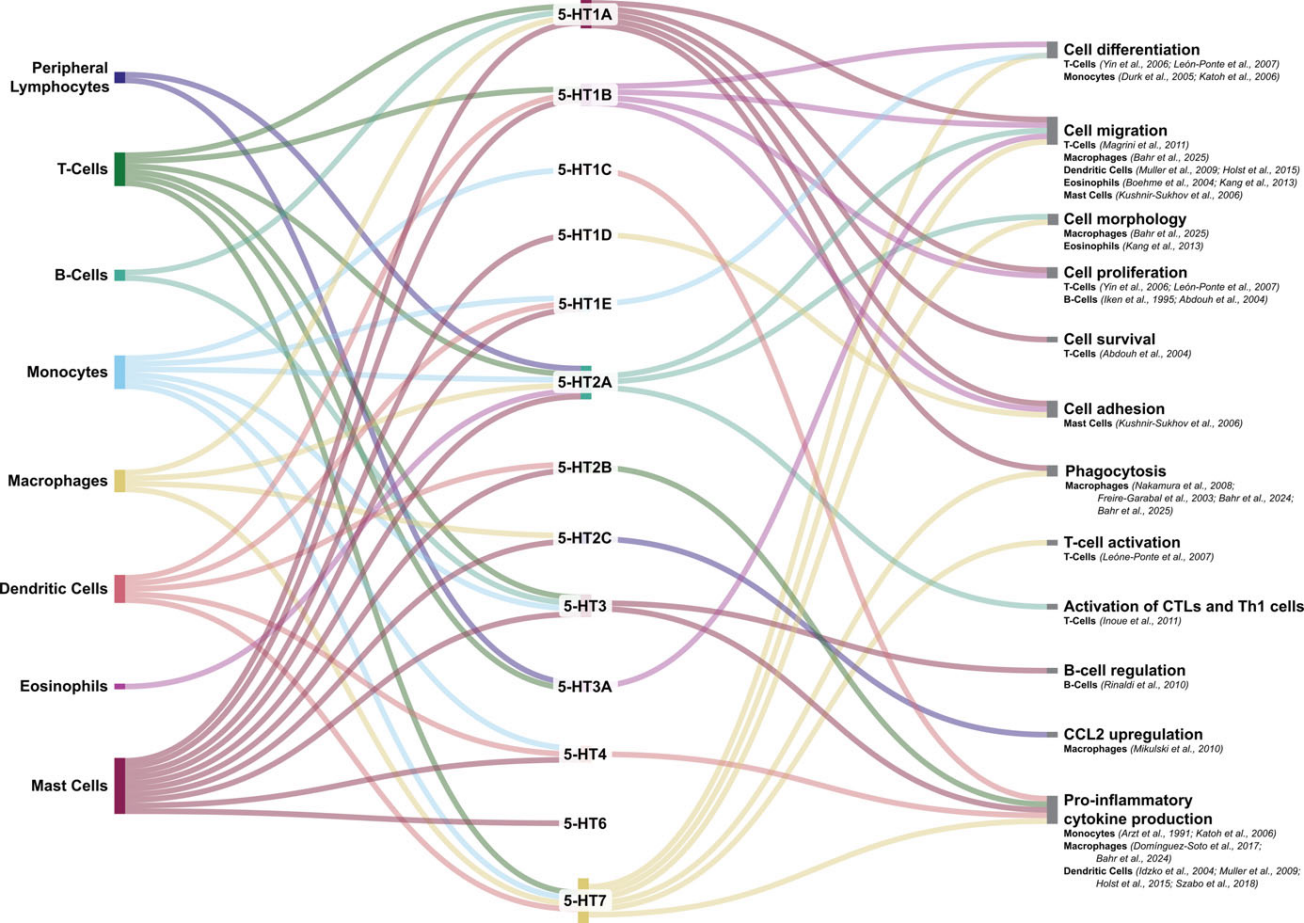
Sankey diagram depicting 5-HT receptor expression in peripheral immune cells and their associated cellular functions. Function-specific references are provided for each cell type. Second messengers and their targets are identified for specific receptors in certain immune cells: **5-HT**_**2A**_ **R** in Eos is associated to increased intracellular Ca^2+^ and to the activation of ROCK, MAPK, PI3K, PKC, and Calmodulin (Boehme *et al*., 2004; Kang *et al*., 2013), **5-HT**_**1A**_ **R** in both T- and B-cells has been associated to NF-κB translocation (Abdouh *et al*., 2004), **5-HT**_**7**_ **R** in T-cells has been found associated to the activation of ERK1/2 and translocation of NF-κB (León-Ponte *et al*., 2007), **5-HT**_**2C**_ **R** in macrophages has been associated to increased intracellular Ca^2+^ (Mikulski *et al*., 2010), and **5-HT**_**4**_ **R** and **5-HT**_**7**_ **R** in dendritic cells have been associated to increases in cAMP levels (Muller *et al*., 2009).

5-HT is known to play a versatile role within the immune system influencing macrophage and monocyte activity, dendritic cell maturation, and natural killer (NK) cell cytotoxicity (Herr *et al*., 2017; Roumier *et al*., 2019). By binding to various 5-HT receptors, 5-HT can either promote or suppress immune responses via the modulation of cytokine production and release. 5-HT receptors are abundantly expressed by immune cells [see Hodo *et al*. (2020) for a thorough review on expression patterns and functions of each receptor]. Figure 2 summarises the expression pattern for 5-HT receptors in peripheral immune cells and associated functions.

Previous *in vitro* studies demonstrate that exogenous 5-HT regulates the release of pro- and anti-inflammatory cytokines and chemokines (e.g., TNF-α) through 5-HT_3_R, 5-HT_4_R, and 5-HT_7_R activation in lipopolysaccharide (LPS)-stimulated peripheral blood mononuclear cell (PBMC) cultures and whole blood (Kubera *et al*., 2000; Cloez-Tayarani *et al*., 2003; Durk *et al*., 2005; Kubera *et al*., 2005). Exogenous 5-HT also promoted anti-inflammatory mechanisms in splenocytes, lymph nodes, and PBMC cultures (Toh and Miossec, 2007; Chabbi-Achengli *et al*., 2016; Sacramento *et al*., 2018). In contrast, exogenous 5-HT impaired the ability of mouse-derived dendritic cells (DCs) to induce Type 1 regulatory (T-reg1) cells and reduced expression of the anti-inflammatory cytokine IL-10 (Liao *et al*., 2023).

*In vitro* evidence also suggests that eosinophils can be modulated by 5-HT. Indeed, treatment with 5-HT caused human eosinophils to roll onto vascular cell adhesion molecule (Vcam)-1, a process important for immune cell migration (Kang *et al*., 2013). This effect was found to be driven by 5-HT_2A_R activation and was coupled with an increase in intracellular calcium (Ca^2+^) levels and with distinct changes in the cytoskeleton and cell shape.

These observations suggest that 5-HT has different cell-specific effects on the immune system, and that these immunomodulatory effects are dependent on various 5-HT receptors.

Immunomodulatory effects have also been observed with commonly prescribed selective 5-HT reuptake inhibitors (SSRIs) and 5-HT and noradrenaline reuptake inhibitors (SNRIs) in whole blood (Diamond *et al*., 2006), PBMC (Taler *et al*., 2007), and microglial cultures (Horikawa *et al*., 2010; Tynan *et al*., 2012; Liu *et al*., 2014), and in *in vivo* immune stimulation paradigms (Pellegrino and Bayer, 2000; Dong *et al*., 2016; Tomaz *et al*., 2020).

Other mechanisms by which SSRIs might modulate the immune system include signalling pathways such as extracellular signal-regulated-protein kinase (Erk) and p38 MAPK cascades (Russo-Neustadt *et al*., 2004; Mercier *et al*., 2004; Chilmonczyk *et al*., 2017), cAMP production (Zhou *et al*., 2016), membrane-associated lipid rafts (Singh *et al*., 2018), glucocorticoid receptors (Pariante *et al*., 2001; Antonioli *et al*., 2012; Gobin *et al*., 2014), the brain derived neurotrophic factor (BDNF) (Wang *et al*., 2022), and the Sigma-1 receptor (Nguyen *et al*., 2015; Hashimoto, 2015; Rosen *et al*., 2019; Salaciak and Pytka, 2022), as well as directly modulating the vagal nerve (Ondicova *et al*., 2019). This suggests that pathways independent of the serotonergic system may be involved in the immunomodulatory effects of SSRIs.

Finally, classical psychedelics moderately influence platelet function and immune responses via serotonin 5-HT2A receptor pathways on platelets and other immune cells but lack significant clotting risks (Szabo, 2015). In contrast, MDMA can markedly disrupt hemostasis, triggering coagulopathies like thrombocytopenia and disseminated intravascular coagulation (DIC) through serotonin syndrome and rhabdomyolysis, highlighting critical safety concerns in therapeutic use (Szabo, 2015; Doyle *et al*., 2020).

While preclinical models provide valuable insights into 5-HT’s immunomodulatory potential, there are critical interspecies differences in receptor expression patterns and pharmacological responses that are crucial to keep in mind. For example, the 5-HT_6_R is abundantly expressed in human and rat striatal regions, however it shows negligible expression in mouse brain tissue (Hirst *et al*., 2003; Kirkpatrick, 2004). Receptor structural variations render many compounds targeting 5-HT_6_R ineffective in murine models (Hirst *et al*., 2003; Kirkpatrick, 2004). These variations are relevant for studying serotonergic psychedelics, as demonstrated by Haberzettl *et al*. (2013) in their systematic analysis of serotonin syndrome models. Their work revealed that monoamine oxidase (MAO)-A knockout mice exhibit greater sensitivity to 5-HT-enhancing drugs compared to wild-type strains, whereas human MAO polymorphisms show more nuanced clinical manifestations (Haberzettl *et al*., 2013; Chiew and Isbister, 2024). This difference originates not only from receptor diversity but also from differences in systemic 5-HT homeostasis and storage mechanisms (Mossner and Lesch, 1998). When considering neuroimmune interactions, physiological disparities complicate translation, particularly given that psychedelics like LSD demonstrate species-specific binding kinetics at the 5-HT_2A_R critical for both psychoactive and immunomodulatory effects (Szabo, 2015; Canal, 2018). These findings underscore the necessity of validating preclinical observations in human cell systems (Figure 2).

### Effect of the immune system on serotonergic transmission

Systemic inflammation has consistently been demonstrated to influence serotonergic signalling in the brain. Systemic LPS injection had a wide range of effects on the brain including increased reuptake of 5-HT from the synaptic cleft via SERT in the mPFC in mice (van Heesch *et al*., 2014), reduced 5-HT concentrations in the hippocampus in mice (Zhao *et al*., 2019) and in the anteroventral preoptic region in rats (Mota *et al*., 2017), increased 5-HT_2A_R mRNA levels in the PFC and hippocampus in mice (Couch *et al*., 2015), increased the functional response to (*R*)-DOI (Couch *et al*., 2015), and increased 5-HT turnover in the PFC and hippocampus in mice (Swiergiel and Dunn, 2006).

In rats treated with the immune stimulus and toll-like receptor (TLR)-3 agonist polyinosinic:polycytidylic acid [Poly(I:C)], levels of messenger RNA (mRNA) coding for SERT increased in the cortex, cerebellum, medial preoptic area, and paraventricular hypothalamic nucleus, which in turn was associated with a decrease in extracellular 5-HT concentrations as measured by microdialysis (Katafuchi *et al*., 2005).

In zebrafish, intra-cerebroventricular (ICV) microinjection of IL-4 suppressed 5-HT production, whereas ICV microinjection of 5-HT suppressed neurogenesis in periventricular neurons via a neuron-glia interaction involving the induction of BDNF (Bhattarai *et al*., 2020).

Systemic LPS injection also leads to induction of the kynurenine pathway in the hippocampus by increasing the expression of the indoleamine-2,3-dioxygenase (IDO) enzyme involved in tryptophan metabolism, leading to an increase in the production of kynurenine from tryptophan (Zhao *et al*., 2019; Marx *et al*., 2021). As tryptophan is an essential amino acid required for the biosynthesis of 5-HT, it has been proposed that IDO induction may limit the availability of tryptophan for 5-HT biosynthesis.

## 5-HT_2A_R psychedelic modulation of immune responses in isolated systems

### Effects of 5-HT_2A_R psychedelics on peripheral blood mononuclear cells

In LPS or Poly(I:C)-stimulated primary monocyte-derived dendritic cells (moDC), *N,N*-DMT, and the methoxylated derivative 5-methoxy-*N,N*-dimethyltryptamine (5-MeO-DMT) inhibit the production of pro-inflammatory cytokines IL-1β, IL-6, and TNF-α and the chemokine IL-8, while increasing the secretion of the anti-inflammatory cytokine IL-10 (Szabo *et al*., 2014). This treatment also reduced the amount of induced T helper (Th)-1 and Th17 effector T-cells versus vehicle in a SIG-1R-dependent manner. Considering that both compounds are endogenous ligands of SIG-1R, the authors propose that activation of the SIG-1R in peripheral immune cells with psychedelics like *N,N*-DMT may trigger a unique cluster of differentiation (CD)4^+^ T-cell response upon viral or bacterial stimulation.

In CD4^+^ and CD8^+^ T-cell cultures stimulated with concanavalin A (ConA), a lectin mitogen, (*R*)-DOI was found to provoke a stimulatory response with an increase in the production of cytokines interferon (IFN)-γ and IL-2 (Inoue *et al*., 2011). This effect was reversed by the 5-HT_2A_R antagonist sarpogrelate hydrochloride. This study found higher doses of (*R*)-DOI to enhance IL-2 and IFN-γ production, indicating a link between 5-HT_2A_R activity and IFN-γ production. The use of ConA, known to induce a distinct anti-cancer T-cell response, might explain the observed difference compared to other studies (Wiersma, 2020).

In primary cultures of CD4^+^ and CD8^+^ T-cells stimulated with anti-human CD3, a widely used T-cell stimulant, levels of TNF-α were unaffected by LSD, *N,N*-DMT, psilocin, or mescaline (Rudin *et al*., 2023). The authors suggest that an initial anti-inflammatory cortisol release upon administration of psychedelics may be responsible for the effects measured in previous investigations (Dos Santos *et al*., 2011) which examined blood and serum samples following administration of psychedelics. Furthermore, cell cultures in this study were treated for 24 to 72 hours which may miss the initial window of immunomodulatory action. Notably, the peak anti-proliferative effects of ayahuasca – a psychoactive decoction containing *N,N*-DMT and monoamine oxidase inhibitors – on CD3+, CD4+, and CD8+ cell populations begin to diminish two hours after administration (Dos Santos *et al*., 2011).

In cultured RAW 264.7 macrophages, Nkadimeng *et al*. (2020) demonstrated that psilocybin-containing mushroom extracts significantly reduced the expression levels of IL-1β following LPS stimulation. Similarly, Oppong-Damoah *et al*. (2019) observed that macrophages stimulated with ethanol produce NO, an effect that was reduced by (*R*)-DOI.

Ghasemi Gojani *et al*. (2024) found that a concentration of 15 μM psilocybin was sufficient to inhibit NLRP3 inflammasome activation and downregulate inflammatory-regulating transcription factors in LPS-stimulated human monocytes. Both 15 and 10 μM doses resulted in the downregulation of NF-κB, Tyrosine Kinase 2 (TYK2), Signal Transducer and Activator of Transcription (STAT)-1, and STAT3 activation – key regulators of IL-1β, IL-6, TNF-α, and cyclooxygenase (COX)-2. This was accompanied by a reduction of COX-2, Pro-TNFα, IL-1β, IL-6, and Pro-IL-1β levels, and suppression of IL-1β release. Interestingly, the 5 μM dose stimulated an increase in the levels of these transcription factors. Higher doses of psilocybin did not alter total NF-κB levels but prevented its nuclear translocation, thereby inhibiting transcription of the regulated genes. In the cases of IL-6 and COX-2, protein levels – but not mRNA levels – were reduced, suggesting that psilocybin may modulate post-translational processing or modifications. This study also found that the LPS-stimulation decreased protein levels of 5-HT_2A_ and 5-HT_2B_ receptors while psilocybin attenuated this downregulation in LPS-stimulated cells, upregulating 5-HT_2A_ receptors above baseline at the highest dose. In unstimulated cells, psilocybin reduced 5-HT_2A_ receptors in a dose-dependent fashion. This research emphasises the immune-modifying capabilities of psilocybin, while also revealing distinct outcomes when applied to inflamed versus non-inflamed biological systems.

Psilocybin inducing opposing immunoregulatory effects at low compared to higher doses has also been observed in resting macrophages, with lower doses inducing higher levels of TNF-α (Laabi *et al*., 2024). Whereas in LPS-stimulated and classically activated macrophages, post-treatment with psilocin, but not psilocybin, produced anti-inflammatory-like effects, reducing levels of TNFα, and increasing levels of IL-10 (Laabi *et al*., 2024). Peyote extract, containing the phenethylamine psychedelic mescaline, activated nitric oxide (NO) production by murine macrophages (Franco-Molina *et al*., 2003). This study further reported that peyote extract stimulated murine thymic lymphocyte proliferation and induced an increase in mRNA levels of IL-1, IL-6, and IL-8 in human leukocytes.

Tourino *et al*. (2013) aimed to assess the effects of *N,N*-DMT and tryptamine (TRY) on IDO activity and the subsequent production of kynurenine. They found that both compounds acted as classical non-competitive inhibitors of IDO. *N,N*-DMT and TRY also increased cytotoxic activity in co-culture assays of A172 glioblastoma cells with PBMCs, suggesting that IDO inhibition by these compounds contributes to a more effective tumour-reactive response by PBMCs. IDO inhibitors have previously been found to produce antidepressant-like properties in animal models of sickness behaviour (O’Farrell and Harkin, 2017; O’Connor *et al*., 2009).

These findings highlight the potential therapeutic application of psychedelics like *N,N*-DMT in enhancing immune responses against tumours, warranting further investigation into their use in cancer immunotherapy.

### Effects of 5-HT_2A_R psychedelics on microglia

In LPS-stimulated microglial cultures derived from mice, *N,N*-DMT and psilocybin reduce expression levels of TLR4 (Kozłowska *et al*., 2021). This suggests their potential to dampen the inflammatory cascade. Additionally, treatment with *N,N*-DMT and psilocybin altered microglial morphology, with treated cells exhibiting a more rounded and compact shape compared to controls. Furthermore, both drugs decreased the expression of co-stimulatory T-cell molecule CD80 and NF-κβ protein, suggestive of a decreased ability of presenting antigens to T-cells. Interestingly, treatment with psilocybin, but not *N,N*-DMT, reduced phagocytosis of healthy neurons by LPS-stimulated microglia, suggesting increased neuroprotection. Additionally, psilocybin upregulated a regulator of microglial phagocytosis and synaptic pruning, Triggering Receptor Expressed on Myeloid cells 2 (TREM2). Mutations in TREM2 have been associated to various neurodegenerative diseases including Alzheimer’s disease and Parkinson’s disease, and its pathway is implicated in anti-inflammatory and neuroprotective functions. If psilocybin upregulates a functional form of TREM2, this could produce potent anti-inflammatory effects, decreasing expression of pro-inflammatory proteins, thus ameliorating disease outcomes.

Proteome analysis of cerebral organoids treated with 5-MeO-DMT found significant alterations in genes associated with long term potentiation, dendritic spine formation, cellular protrusion formation, microtubule and cytoskeletal organisation, and mild activation of T lymphocyte differentiation (Dakic *et al*., 2017). Following 5-MeO-DMT treatment, they also observed downregulation in protein expression of pathways associated to nuclear factor of activated T-cells (NFAT) and NF-κB signalling via TLR- and Gq-coupled receptors.

In TNF-α and IFN-γ stimulated C6-glioma cells, (*R*)-DOI produced a dose-dependent inhibition of cytokine-induced NO levels (Miller and Gonzalez, 1998). This effect was not seen when (*R*)-DOI was added more than 2 hours after the immune stimulation was added. This suggests that (*R*)-DOI suppression of NO occurs at a transcription regulation level.

Another study looking at NO release in LPS and IFN-γ stimulated BV-2 murine microglia showed that pre-treatment with psilocin reduced NO release. This effect was dependent on the 5-HT_2A_R, as inhibiting it with the antagonists cyproheptadine and risperidone prevents the reduction in NO release seen by psilocin (Wiens *et al*., 2024). Additionally, this study demonstrated that psilocin could reduce levels of ROS in human microglia-like cells primed with LPS and stimulated with the bacterial peptide N-formyl-Met-Leu-Phe (fMLP) (Wiens *et al*., 2024). Increases in ROS have been seen in clinical studies and preclinical models of neuropsychiatric disorders like major depressive disorder (MDD) and are also believed to play a role in the pathogenesis of neurodegenerative diseases (Simpson and Oliver, 2020; Rossetti *et al*., 2020). The capability of psychedelics to reduce the production of ROS might be one of the mechanisms with which they modulate the inflammatory response.

Although the research reviewed so far in cell cultures or organoids indicate immunomodulatory properties of some 5-HT_2A_R psychedelics, there is no clear consensus on whether psychedelics are pro- or anti-inflammatory. Additional research is warranted to definitively establish whether psychedelics can directly affect PBMC proliferation and function, microglial activation, and the production and release of cytokines *in vivo* and the mechanisms underlying such effects.

### Immune organs and psychedelics

In LPS-stimulated mouse splenic cell cultures, low concentrations of LSD enhanced both baseline and IL-2-augmented NK cell function, but higher doses suppressed the NK response (House *et al*., 1994). These high LSD doses also led to suppression of B-cell function, macrophage function, reduced numbers of cytotoxic lymphocytes, and a reduction in Th1 cell IL-2 production.

In rat primary aortic smooth muscle cells treated with TNF-α, pre-treatment and co-treatment with (*R*)-DOI for 24 hours inhibited the TNF-α-induced inflammatory responses in a 5-HT_2A_R-dependent fashion (Yu *et al*., 2008). (*R*)-DOI potently suppressed the expression of key inflammatory mediators, notably the cytokine IL-6, the intracellular adhesion molecules ICAM-1 and VCAM-1, important components of atherosclerotic plaque formation, compared to TNF-α stimulated cells alone. Notably, (*R*)-DOI by itself did not elicit any response. Furthermore, the authors showed similar inhibitory effects on TNF-α induced inflammation with other 5-HT_2A_R agonists, including a phenethylamine [(4-bromo-3,6-dimethoxybenzocyclobuten-1-yl) methylamine (2C-BCB)], and two indolealkylamines, lysergic acid 2,4-dimethylazetidide (LA-SS-Az) and LSD, suggesting a broader class effect.

Psilocybin was found to reduce expression levels of TNFα, IFN-γ, IL-6 and IL-8, in a human 3D epi-Intestinal tissue model treated with TFN-α/IFN-γ, providing additional evidence for the anti-inflammatory properties of psychedelics (Robinson *et al*., 2023).

The studies mentioned so far have used a range of concentrations of psychedelics. The clinically relevant psychedelics psilocybin, *N*,*N*-DMT, 5-MeO-DMT, and LSD were all used in supraphysiological concentrations (House *et al*., 1994; Kozłowska *et al*., 2021; Szabo *et al*., 2014; Rudin *et al*., 2023), meaning that the doses used may have exceeded circulating concentrations as observed in clinical trials of psychedelics for the treatment of MDD (Ross *et al*., 2016; Carhart-Harris *et al*., 2017; Carhart-Harris *et al*., 2018; Doss *et al*., 2021; Carhart-Harris *et al*., 2021; Goodwin *et al*., 2022, 2023a, 2023c;Raison *et al*., 2023).

### Receptors mediating the immunomodulatory effects of 5-HT_2A_R psychedelics

Although the involvement of the 5-HT_2A_R in the immunomodulatory effects of psychedelics has not yet been fully elucidated, the studies explored so far have reported anti-inflammatory effects which appear to be 5-HT_2A_R-dependent (Yu *et al*., 2008; Nau *et al*., 2013). The 5-HT_2A_R is likely the major binding site which is most closely associated with the anti-inflammatory effects observed in preclinical studies. However, 5-HT_2A_R psychedelics have been reported to bind other receptors.

#### Sigma-1 receptor

Alongside *N,N*-DMT, psilocybin and the non-classical psychedelic ibogaine are hypothesised to bind Sig-1R in the CNS and on circulating immune cells (Sershen *et al*., 1996; Mason *et al*., 2023). Sig-1R is highly enriched at the mitochondria-associated endoplasmic reticulum (ER) membrane and is expressed in immune cells (Zhang *et al*., 2023a), and throughout the CNS (Shi *et al*., 2021). It is believed to exert neuroprotective effects by suppressing ER stress, regulating Ca^2+^ influx into mitochondria and adenosine triphosphate (ATP) synthesis, preventing excitotoxicity and oxidative stress by regulating key ER membrane proteins and downstream transcription factors NF-κB and X-box binding protein 1 (XBP-1) (Hayashi and Su, 2007; Ho *et al*., 2018; Hayashi, 2019). Additionally, Sig-1R plays crucial roles in neuronal differentiation, cell survival, and immune modulation, promoting anti-inflammatory actions and BDNF secretion, as well as regulating neuroplasticity and glial reactivity in rodent models (Peviani *et al*., 2014; Ruiz-Cantero *et al*., 2021).

Overexpression of astrocytic Sig-1R using an adeno-associated virus in cultured primary astrocytes was found to attenuate LPS-driven IL-1β, TNF-α, and inducible nitric oxide synthase (iNOS) production, while increasing BDNF production and reducing astrocyte and microglial activation. Overexpression of astrocytic Sig-1R in a mouse model also reduced LPS-induced depressive-like behaviour and improved memory function (Guo *et al*., 2021).

Macrophages and microglia activation states have been organised in a spectrum based on protein expression ranging from an M1 pro-inflammatory state to an M2 anti-inflammatory state. Activation of Sig-1R promotes the microglial M2 state while inhibiting the M1 state, and promotes astrocytic glial fibrillary acidic protein (GFAP) expression and BDNF secretion in response to inflammatory stimuli (Jia *et al*., 2018).

It has been reported that Sig-1R activation suppresses the ability of microglia to rearrange their actin cytoskeleton, migrate, and release cytokines in response to ATP, monocyte chemoattractant protein 1 (MCP-1), and LPS in primary glial cultures (Hall *et al*., 2009). In the same study, it was observed that stimulation of Sig-1R suppressed both transient and sustained intracellular Ca^2+^ elevations associated with the microglial response to these activators. Furthermore Sig-1R activation suppressed membrane ruffling, preventing microglial migration and stress-induced actin reorganisation in the cell, in a Ca^2+^-independent manner, suggesting that interactions between microglia and the Sig-1R may be multifaceted.

Shen *et al*. (2008) assessed treatment with the Sig-1R agonist dimemorfan in an inflammatory ischaemic stroke model in rats. They observed inhibited expression of MCP-1 and IL-1β, decreased neutrophil infiltration, decreased activation of p38 MAPK, NF-κB, and STAT1, and decreased expression of neuronal and inducible NOS in the cortex. These changes were further attributed to decreased extracellular glutamate accumulation. Rather than directly modulating microglia, Sig-1R might be influencing microglial function indirectly by regulating glutamate concentrations extrasynaptically.

#### Tropomyosin receptor kinase B receptor

5-HT_2A_R psychedelics might also influence the immune system by interacting with Tropomyosin receptor kinase B (TrkB) receptors, to which BDNF and other neurotrophins can bind. Neurotrophins are signalling molecules primarily found in the peripheral and CNS and are primarily stored in platelets with platelet concentrations reaching up to 1000 times the concentrations found in neurons (Boukhatem *et al*., 2021). TrkB mediates processes such as synaptogenesis, neuroplasticity, apoptosis, mammalian target of rapamycin (mTOR) signalling pathway activation, and phospholipase C gamma (PLC-γ1) activity (Colle *et al*., 2015; Zhang *et al*., 2016).

It has also been implicated in the regulation of the immune system. Subsets of T-cells, macrophages, and DCs express TrkB receptors (Ciriaco *et al*., 1996; De Santi *et al*., 2009; Kozlov *et al*., 2020). BDNF is produced by CD4^+^ and CD8^+^ T-cells, B-cells, and monocytes, and has been shown to promote neuronal survival *in vitro* (Kerschensteiner *et al*., 1999). Reduced TrkB signalling and BDNF signalling in the brain are observed in response to LPS administration and Poly(I:C) administration in rodents (Gibney *et al*., 2013), as well as increased depressive-like behaviour, which can be rescued by administration of a TrkB agonist (Zhang *et al*., 2014).

Psilocybin and LSD directly bind to the TrkB receptor with 1000-fold higher affinity than SSRIs (Moliner *et al*., 2023). Psychedelics increase the neuronal surface retention of TrkB and promote BDNF downstream signalling (Moliner *et al*., 2023). This binding increases TrkB interaction with PLC-γ1, a critical regulator of NF-κB, MAPK/ERK signalling, calcium homeostasis via IP_3_, and activated T-cell signalling, via the NFAT family of transcription factors (Tao *et al*., 2023).

While the exact mechanisms are unclear, preliminary studies suggest TrkB might be a key player in how 5-HT_2A_R psychedelics influence the immune system. Psychedelics bind to TrkB with greater affinity than traditional antidepressants, influencing cellular signalling pathways, potentially affecting immune cell function. Further research is needed to fully understand these effects.

## Psychedelic modulation of immune responses in whole systems

Isolated systems cannot fully capture the interactions between different tissues and organs within a whole organism. Preclinical studies in animals and clinical studies are crucial to determine whether 5-HT_2A_R psychedelics directly impact the immune system or exert an indirect immunomodulatory effect mediated by the CNS, the HPA or sympathetic-adrenal-medullary (SAM) axes, or other cellular pathways. These *in vivo* whole system investigations provide insights into how psychedelics interact with the immune system and shed light on the translational potential of 5-HT_2A_R psychedelics for immune modulation. This section reviews recent studies assessing the immune system’s response to 5-HT_2A_R psychedelics conducted in animal models and in human clinical trials.

### Animal models

Preclinical models are not perfect representations of human diseases; however, they offer many advantages to elucidate the mechanisms underlying the effect of psychedelics on the CNS and the immune system. These advantages include a controlled environment to isolate specific factors, the accessibility of cells and tissues at different life stages to track progression, genetic manipulation, and the means to test experimental drugs on bi-directional immune-nervous system interactions. There have been several studies investigating the effect of psilocybin, (*R*)-DOI, LSD, ayahuasca, and novel psychedelics, on immune responses and mediators *in vivo*, in healthy animals as well as in disease models.

In an exploratory study, Bove and Mokler (2022) administered psilocybin to healthy female rats and measured pro- and anti-inflammatory markers in peripheral serum. Psilocybin induced the release of pro-inflammatory factors IL-1β, TNF-α, IL-13, MCP-1, and C-X-C motif chemokine ligand 10 (CXCL10), and anti-inflammatory factors IFN-γ, IL-10, granulocyte colony-stimulating factor (G-CSF), into the serum, consistent with a generalised activation of the immune system. This difference was persistent seven days after psilocybin injection, however, as the authors note, high variability in the results limit the conclusions that can be drawn from this study. In support of this, Custodio *et al*. (2023) reported neurotoxic properties of (*R*)-DOI and other 5-HT_2C_R agonists in healthy mice, with increased expression of ionised calcium binding adaptor molecule 1 (Iba1), IL-6, and TNF-α, however, these effects were reported at very high doses (30 mg/kg).

LSD, administered to healthy mice, was found to reduce hippocampal levels of kynurenine, altering the kynurenine/tryptophan and the kynurenine/5-HT ratios significantly, without altering levels of hippocampal 5-HT (Inserra *et al*., 2023). This highlights psychedelics’ potential therapeutic role in disorders with a dysregulated kynurenine pathway, which is discussed in detail later. The same group also observed modulation of endocannabinoid-related metabolites in the hippocampus following LSD administration. These effects were correlated with increased sociability (Inserra *et al*., 2023).

In the context of disease models, 5-HT_2A_R psychedelics have consistently been reported to suppress the immune response.

Mice injected with sheep erythrocytes – activating both T and B lymphocytes (McAllister *et al*., 2017) – and subsequently treated with (*R*)-DOI exhibited a marked suppression of the immune response (Davydova *et al*., 2010). This was evidenced by a significant decrease in CD8^+^ T-cells, both in peripheral blood and the spleen. Notably, administration of ketanserin, a 5-HT_2A_R antagonist, produced the opposite effect. These findings strongly support the involvement of 5-HT_2A_R in mechanisms leading to immunosuppression.

Nichols and colleagues have been able to demonstrate anti-inflammatory properties for psychedelics specifically via 5-HT_2_ receptor sub-type activation using various animal models. For example, a rapid immune response can be induced after systemic administration of exogenous TNF-α, with an increase in circulating IL-6 and VCAM-1. Administration of (*R*)-DOI efficiently blocks these effects of TNF-α, with reduced IL-6 and VCAM-1 expression in the aortic arch and small intestine (Nau *et al*., 2013). The mechanism underlying this potent anti-inflammatory response appears to be the activation of 5-HT_2A_R.

Ovalbumin (OVA) induced acute allergic asthma in mice provides a model to study immune-modulatory properties of experimental compounds. Using this model, (*R*)-DOI was found to attenuate various asthma parameters in response to a non-selective muscarinic receptor agonist methacholine, including elevated airway hyperresponsiveness and pulmonary inflammation (Flanagan *et al*., 2019a). Interestingly, the authors measured a reduction in expression of certain pro-inflammatory cytokines such as IL-15 and IL-9, but an increase in expression of other pro-inflammatory cytokines IL-13 and IL-33 which are known contributors to a persistent chronic asthma state. In a later study using the OVA model, (*R*)-DOI was found to prevent OVA-induced increases in mRNA levels for pro-inflammatory cytokines IL-5, IL-6, TNF-α and IL-1β (Flanagan *et al*., 2021). The authors identified 2,5-dimethoxyphenethylamine (2C-H) from 21 different 5-HT_2A_ agonists as the key pharmacophore mediating effective anti-inflammatory properties. This may be a useful starting point for future drug development focusing on anti-inflammatory action.

More recently, Flanagan *et al*. (2024) aimed to identify key structural components of 5-HT_2A_R agonists mediating their anti-inflammatory effects focusing on their ability to suppress Arginase-1 (Arg1) expression in peripheral tissues. Arg1 catalyses the conversion of L-arginine into L-ornithine and urea. Upregulation of Arg1 has been shown to contribute to inflammation and airway obstruction (North *et al*., 2009), whereas in the absence of Arg1 activity, L-arginine is instead converted into NO via NOS activity, contributing to relaxation of bronchial smooth muscle and inhibition of inflammation (Cloots *et al*., 2013). Despite having similar *in vitro* activity on 5-HT_2A_R and similar behavioural potency, the novel agonist (*R*)-2,5-dimethoxy-4-trifluoromethylamphetamine [(*R*)-DOTFM] did not exhibit anti-inflammatory properties like (*R*)-DOI. Only (*R*)-DOI led to significant reductions in levels of Arg1, IL-6, and CXCL10. The authors argue that the different effects of (*R*)-DOTFM and (*R*)-DOI may originate from differences in receptor stabilisation and conformation which could lead to separate downstream effectors and pathways being recruited. These experiments are the first to study and identify differences in functional selectivity of 5-HT_2A_R agonists in peripheral tissues, indicating molecular and cellular sensitivities underlying anti-inflammatory properties of serotonergic 5-HT_2A_R psychedelics. This may inform future studies to identify novel anti-inflammatory compounds devoid of subjective psychedelic effects.

There are additional animal models that have provided insights into the anti-inflammatory effects of psychedelics. In a mouse model of cardiovascular disease [Apolipoprotein E (ApoE)^-/-^ on a high fat diet], continuous systemic infusion of low-dose (*R*)-DOI resulted in significant reductions in mRNA expression of pro-inflammatory markers, including IL-6, TNF-α and CXCL10 (Flanagan *et al*., 2019b). (*R*)-DOI treatment normalised glucose homeostasis and reduced circulating cholesterol. Although this study is not directly comparable, research in human vascular smooth cell cultures found that serotonin increased IL-6 production through 5-HT_2A_R, suggesting that psychedelics and serotonin may activate different downstream cellular pathways (Ito *et al*., 2000).

Psilocybin has been shown to have anti-inflammatory properties in different models of peripheral inflammation. In mice systemically injected with LPS, psilocybin treatment, pre- or post-administration of LPS, was found to reduce expression of IL-6 and TNF-α cytokines in homogenised brain tissue and in peripheral blood (Zanikov *et al*., 2023). Inflammation can also be induced via the gut-brain-axis by orally administering dextran sulphate sodium (DSS) to mice or rats to induce acute colitis. This has been shown to lead to immune responses in the gastrointestinal tract as well as in the CNS (Dempsey *et al*., 2019). In a recent study using this model, post-treatment with psilocybin and eugenol – a positive allosteric modulator of the gamma-aminobutyric acid (GABA)-A receptor – reduced expression of pro-inflammatory cytokines and markers IL-1β, IL-6, and COX-2 in the brain (Zanikov *et al*., 2024). These results demonstrate a clear immunomodulatory property of psilocybin in the context of induced systemic inflammation.

Streptozotocin (STZ), a glucosamine–nitrosourea compound leading to an insulin-resistant brain state, has been previously used to induce an Alzheimer’s disease model in the rat as intraperitoneal or intracerebroventricular injection of STZ leads to production of amyloid-beta (Kadhim *et al*., 2022). Using this model, Afshar *et al*. (2019) reported that both a selective antagonist of 5-HT_1A_R (NAD-299) and a selective agonist of 5-HT_2A_R (TCB-2) reduced oxidative stress in the hippocampus and provided neuroprotection. In support of this, a separate study using an Aβ-induced mouse model of AD found that *N,N*-DMT alleviated astrocytic activation and astrogliosis in the hippocampus and dentate gyrus measured via GFAP immunostaining (Borbély *et al*., 2022).

*N,N*-DMT administration also produced anti-inflammatory and pro-neurotrophic effects in a rat ischaemic brain injury model. Pretreatment with *N,N*-DMT led to lower expression of both mRNA and protein levels of a key activator of the apoptotic cascade (Apoptotic Protease Activating Factor 1 – APAF-1), and higher BNDF expression in this model, as well as a reduction in TNF-α, IL-1β, IL-6 and an increase in IL-10 levels (Nardai *et al*., 2020).

Szabo *et al*. (2021) also demonstrated a neuroprotective effect in the ischaemic rat brain following *N,N*-DMT administration, with a reduction in the number of apoptotic cells and improved astrocytic survival. However, they demonstrated this effect to be mediated by Sig-1R activation. In a more recent study, pre-treatment with psilocybin reduced brain infarction and neurological deficits following induced stroke in rats, whereas post-treatment also led to downregulation of Iba1 (Yu *et al*., 2024). These effects were attenuated by the BDNF inhibitor and TrkB antagonist ANA12.

A recent study used caecal ligation and puncture to model sepsis in rats and assess how ayahuasca might produce anti-inflammatory and neuroprotective effects (de Camargo *et al*., 2024). Ayahuasca pre-treatment increased levels of IL-4 and BDNF in the cortex, while enhancing neutrophil activation and decreasing nitric oxide signalling. At a behavioural level, ayahuasca reduced anxiety-like measures in behavioural tests, suggesting that it can prevent sepsis-induced neuroinflammatory and oxidative stress, and reduce anxiety-like behaviour.

Overall, these results suggest that modulation of serotonin receptors, and possibly (SIG-1R), offer protection against neuroinflammation induced by various disease salient stimuli, highlighting the potential role of the serotonergic system and the Sig-1R in inflammation associated neurodegenerative processes.

Studies have also found that psychedelics modulate the expression of immunological factors reported to be increased in stress associated behavioural paradigms. Kelley *et al*. (2022) measured an increased expression of IL-1β and its receptor IL1r1 in a rat model for post-traumatic stress disorder in the prefrontal cortex. They found that IL1r1 was significantly reduced after treatment with *N,N*-DMT, or a combination of *N,N*-DMT and harmaline, as well as TLR4, TLR6, and TLR7 (Kelley *et al*., 2022). In response to repeated social aggression, stressed mice exhibit elevated cytokine gene expression and increased TNFα levels in plasma and CSF (Krupp *et al*., 2024). A single administration of (*R*)-DOI after stress induction reduced plasma and limbic brain levels of TNFα and promoted escape, a dynamic coping strategy, indicating anxiolytic effects.

This emerging evidence suggests potent immunomodulatory properties of classical psychedelics, particularly (*R*)-DOI and psilocybin. *In vivo* studies in both mice and rats have demonstrated that 5-HT_2A_R agonists can influence responses in immune-challenge models via a suppression of factors associated with the inflammatory response system (Nau *et al*., 2013; Flanagan *et al*., 2019a, b, 2021; Nardai *et al*., 2020; Zanikov *et al*., 2023), a decrease in CD8^+^ T-cells proportion (Davydova *et al*., 2010), and a reduction in oxidative stress (Afshar *et al*., 2019; Szabo *et al*., 2021). Interestingly, as referred to earlier, psilocybin led to a general activation of the immune system with a release of pro- and anti-inflammatory cytokines in healthy unstimulated rats, suggesting possibly different responses based on activation state (Bove and Mokler, 2022). Similarly, (*R*)-DOI treatment has been associated with reduced response induced in the OVA model, such as a significant reduction in mucus production and pulmonary inflammation (Flanagan *et al*., 2019a), and normalised glucose homeostasis in the ApoE^-/-^ model, in mice (Flanagan *et al*., 2019b).

According to these preclinical *in vivo* studies, psychedelics appear to have a dual effect on the immune system. Under normal conditions, without external triggers, psychedelics tend to stimulate immune system activity. However, when the immune system is already activated by external factors, such as with LPS administration, psychedelics seem to inhibit the immune response to these triggers. This suggests that psychedelics may have a regulatory effect on immune function, potentially stimulating or suppressing immune activity depending on the body’s current state.

### Human studies

Following administration of a high dose of psilocybin in healthy volunteers, Hasler *et al*. (2004) observed a significant rise in thyroid–stimulating hormone (TSH), prolactin, adrenocorticotropic hormone (ACTH), and cortisol. Interestingly, this increased hormonal release wasn’t associated with heightened anxiety. The authors suggest this observation aligns with known effects of 5-HT_2_ receptor stimulation, which can trigger the HPA axis, leading to elevated ACTH and cortisol levels. Given that cortisol is known for its anti-inflammatory properties (Rhen and Cidlowski, 2005), it might be expected that elevated cortisol levels could influence inflammatory markers. Conversely, Burmester *et al*. (2023) reported no change in circulating inflammatory markers [C-reactive protein (CRP) and TNF-α] after a single psilocybin dose in 16 healthy participants. Similarly, four weeks after a psilocybin dose, a transient increase in peripheral cytokine production was reported. However, this increase was not consistent across different patient populations which included healthy participants, patients with depression, anxiety, and cancers of various types (DiRenzo *et al*., 2024).

Studies on *N,N*-DMT, a key component of ayahuasca, indicate its potential ability to acutely influence neuroendocrine and immune markers. After *N,N*-DMT administration using freeze-dried ayahuasca capsules, Dos Santos *et al*. (2011) observed decreased CD4^+^ and CD3^+^ cell populations in healthy individuals and those with treatment-resistant depression (TRD), increased proportion of NK cells, and increased prolactin and cortisol levels. Notably, this modulatory effect was transient, with peak effects occurring two hours after intake and returning to baseline within 24 hours. A larger study by Galvao *et al*. (2018) demonstrated a rise in salivary cortisol levels in both healthy controls and patients with TRD during the ayahuasca session compared to placebo, returning to baseline within 48 hours. Interestingly, subsequent research revealed a significant correlation between greater reductions in CRP and lower depressive symptoms at 48 hours post-ayahuasca in both patient and control groups (Galvao-Coelho *et al*., 2020). These findings suggest a potential link between ayahuasca’s antidepressant action and its effect on neuroendocrine and immune systems.

Preliminary evidence also indicates that 5-MeO-DMT has immunomodulatory properties. In experienced psychedelic users, a significant decrease in salivary IL-6 and a rise in cortisol was measured after 5-MeO-DMT inhalation (Uthaug *et al*., 2020). However, further investigation is required as this study lacked a suitable placebo, had a small sample size, and used variable dosing. 5-MeO-DMT is similarly active at 5-HT_2A_ and 5-HT_1A_ receptors and binds to 5-HT_1A_R as a biased agonist, similar to the partial agonist and anxiolytic buspirone (Warren *et al*., 2024).

**Table 1.** Summary of *in vitro* studies having measured immunomodulatory properties of 5-HT_2A_R psychedelics

Cell type	Stimulus	Psychedelic concentration	Immune effect measured	Results	Putative pathway	References
Psilocybin-containing mushroom						
RAW 264.7 macrophages	LPS	[10–50] μg/mL	Cytokine, NO, Prostaglandin E2 production	↓ Expression levels of IL1B, NO and PGE2	Antioxidant activity	Nkadimeng *et al*. (2020)
Psilocybin or psilocin						
Mouse primary CD11b+ microglia	LPS	100 μM	• Protein expression of TLR4, NF-κB, and CD80 • Expression of TREM2 • Microglial phagocytosis	↓ TLR4, p65, and CD80 levels ↑ TREM2 levels • Attenuation of healthy neuron phagocytosis by microglia • Changes in microglial morphology	TLR4 downregulation	Kozłowska *et al*., (2021)
Human 3D EpiIntestinal Tissue	TNF-α/IFN-γ	[10–40] μM	Cytokine protein levels	↓ TNF-α, IFN-γ, IL-6, IL-8 levels	• 5-HT1A, 2A, 2B, 2C signalling • β-arrestin pathway • Glucocorticoid signalling pathways	Robinson *et al*. (2023)
• Primary cultures CD4+, CD8+ T cells • THP1 monocytes	• anti-human CD3 • LPS	[1–30] μM	• TNF-α levels • NF-κB expression	No effect seen		Rudin *et al*. (2023)
Raw 264.7 macrophages	LPS	Psilocybin: [6–24] ng/mL Psilocin: [14–56] ng/mL	Protein levels of TNF-α and IL-10.	Pre- and post-treatment ↓ TNF-α levels Pre-treatment ↓IL-10 levels	5-HT7R signalling or NF-κB	Laabi *et al*. (2024)
Human THP1 monocytes	LPS	[5–15] μM	• NLRP3 inflammasome activation • STAT 1 and STAT3 activation • Cytokine production • 5-HT2A and 5-HT2B receptor expression	↓ NF-κB, STAT 1 and STAT 3 transcription factors ↓ COX-2, IL-6 protein levels ↓ IL-1β and TNF-α mRNA levels • Higher doses of psilocybin prevented nuclear translocation of NF-κB • Attenuation of LPS-induced increase in 5-HT_2A_R and 5-HT_2B_R expression	NF-κB, STAT 1 and STAT3	Ghasemi Gojani *et al*. (2024)
BV-2 murine microglia and human microglia-like cells	LPS, IFN-γ, bacterial peptide	[0.1–10] μM	NO and ROS release	↓ NO release in BV-2 murine microglia ↓ ROS levels in human microglia-like cells	• β-arrestin inhibition of NF-κB • Inhibition of iNOS and NOX • STAT and PKC	Wiens *et al*. (2024)
LSD						
Mouse splenic cell cultures	LPS	[0.0001–100] μM	• NK cell activity • IL-2, IL-4, IL-6 production	• Suppression of NK cell activity at 100 μM • Reduced cytokine protein levels	Cytotoxic T-lymphocyte suppression	House *et al*. (1994)
• Primary cultures CD4+, CD8+ T cells • THP1 monocytes	• anti-human CD3 • LPS	[1–30] μM	• TNF-α levels • NF-κB expression	No effect seen		Rudin *et al*. (2023)
*N,N*-DMT						
Monocyte derived dendritic cells (MoDC)	LPS, Poly I:C, *E. coli*, or influenza virus	100 μM	• Cytokine and chemokine production • Number of induced effector T cells	↓ IL-1β, IL-6, TNF-α and IL-8 ↑ IL-10 ↓ Number of TH1 and TH17 effector T cells	• Sigma1-R • CD4+ T-cell response	Szabo *et al*. (2014)
PBMCs and glioma cell line A172 co-cultures	IFN-γ	100 μM	• IDO expression • Antitumoural activity	↓ IDO mRNA expression ↓ number of tumour cells	Kynurenine pathway	Tourino *et al*. (2013)
• Primary cultures CD4+, CD8+ T cells • THP1 monocytes	• anti-human CD3 • LPS	[1–30] μM	• TNF-α levels • NF-κB expression	No effect seen		Rudin *et al*. (2023)
Mouse primary CD11b+ microglia	LPS	100 μM	• Protein expression of TLR4, NF-κB, and CD80 • Expression of TREM2 • Microglial phagocytosis	↓ TLR4, p65, and CD80 levels ↑ TREM2 levels	TLR4 downregulation	Kozłowska *et al*., (2021)
5-MeO-DMT						
Monocyte derived dendritic cells (MoDC)	LPS, Poly I:C, *E. coli*, or influenza virus	100 μM	• Cytokine and chemokine production • Number of induced effector T cells	↓ IL-1β, IL-6, TNF-α and IL-8 ↑ IL-10 ↓ Number of Th1 and Th17 effector T cells	• Sigma1-R • CD4+ T-cell response	Szabo *et al*. (2014)
• Human neural progenitor cells • Human cerebral organoids	Unstimulated	[23–7000] nM	• T-lymphocyte differentiation • NF-κB and NFAT signalling • Proteome analysis	• Inhibition of NF-κB signalling ↓ mGluR5 expression • Modulation of protein expression involved in long-term potentiation • Modulation of protein expression involved in cytoskeletal reorganisation and dendritic spine morphogenesis	• TLR4 • 5-HT2AR and 5-HT2CR • Sigma1-R	Dakic *et al*. (2017)
(*R*)-DOI						
Rat C6 glioma cells	TNF-α/IFN-γ	1 μM	Cytokine-induced NO levels	• Dose-dependent inhibition of NO levels • Prevented by PKC inhibitor • Effect not seen when (*R*)-DOI was applied 2 hours following stimulation.	5-HT_2A_R signalling and PKC	Miller and Gonzalez (1998)
Rat primary aortic smooth muscle cells	TNF-α	1 nM	Pro-inflammatory cytokine expression	↓ IL-6, ICAM-1, VCAM-1 levels	5-HT_2A_R signalling	Yu *et al*. (2008)
CD4+ and CD8+ cell cultures from BALB/c mice	Concanavalin A	[10^-15^–10^-12^] μM	Cytokine production	↑IFN-γ and IL-2 • Effect reversed by 5-HT_2A_R antagonist sarpogrelate • Contributes to cytotoxic T-lymphocyte and Th1 cell activation	5-HT/5-HT_2A_R signalling	Inoue *et al*. (2011)
• Eos-like cell line AML14.3D10 • Human Eos • Murine Eos	5-HT	10 μM	• Changes in cytoskeleton and morphology • Rolling of cells onto VCAM-1 • Intracellular Ca^2+^ levels • Eos trafficking	• Induced rolling and migration of Eos • Actin polymerisation in AML14.3D10 cells ↑ intracellular Ca^2+^ levels	ROCK, MAPK and PI3K signalling	Kang *et al*. (2013)
Raw 264.7 macrophages	Ethanol	0.3 nM	NO production	↓ NO production	5-HT_2A_R signalling	Oppong-Damoah *et al*. (2019)

**Table 2.** Summary of *in vivo* studies, both in rodents and humans, having measured immunomodulatory properties of 5-HT2AR psychedelics

Species		Stimulus or model	Psychedelic concentration	Immune effect measured	Results	Putative pathway	References
Psilocybin							
Rodents	Female Sprague-Dawley rats	Unstimulated	20 mg/kg intraperitoneally	Serum levels of cytokines and chemokines	↑ IL-1β, IL-10, IL-13, IFN-γ, IP-10, G-CSF, MCP-1, TNF-α, and leptin	5-HT_2A_R signalling	Bove and Mokler (2022)
	C57BL/6J mice	LPS	0.88 mg/kg gavage	• Pro-inflammatory cytokine protein levels • Gene expression in brain and peripheral blood	↓ Expression and protein levels of TNF-α and IL-6	5-HT_2A_R signalling	Zanikov *et al*. (2023)
	C57BL/6J male mice	Unstimulated	[0.3–3] mg/kg intraperitoneally	Plasma corticosterone	Elevations in plasma corticosterone and anxiolytic-like effects which are blunted by pre-treatment with glucocorticoid receptor antagonist but not 5-HT_2A_R antagonist.	Psilocybin induced glucocorticoid release	Jones *et al*. (2023)
	C57BL/6J mice	Dextran Sulphate Sodium-induced colitis	0.88 mg/kg gavage	• Pro-inflammatory cytokine protein levels • Gene expression in brain and peripheral blood	↓ Protein levels of IL-2 and IL-1β gene mRNA expression in the brain	Gut-brain axis modulation	Zanikov *et al*. (2024)
	Male Sprague-Dawley rats	Middle cerebral artery occlusion	100 µM × 20 µL intracerebroventricularly	• Brain infarction region • Neurological deficits • IBA1 microglial expression	↓ Brain infarction and neurological deficits and IBA1 expression • Effects attenuated by ANA-12 (TrkB receptor antagonist)	TrkB receptor	Yu *et al*. (2024)
	C57BL/6J mice	Restraint stress	5 mg/kg intraperitoneally	• Plasma corticosterone levels • Depressive- and anxiety-like behaviour	↑ plasma corticosterone in both sexes in response to psilocybin, no additive effects with stress model. Reduction in anxiety-like behaviour	HPA axis	Farinha-Ferreira *et al*. (2025)
Humans	Male and female humans	Unstimulated	[45–315] μg/kg orally	TSH, prolactin, ACTH and cortisol levels	↑ TSH, prolactin, ACTH and cortisol plasma levels	HPA axis modulation, endocrine modulation	Hasler *et al*. (2004)
	Male and female humans	Unstimulated	0.22 mg/kg orally	CRP serum and TNF-α plasma levels	No effect seen	n/a	Burmester *et al*. (2023)
	Male and female humans	Healthy participants or patients with depression, anxiety, or cancer	Range of doses	Peripheral cytokines	Transient increase in peripheral cytokines, although not consistent across the different patient populations	n/a	DiRenzo *et al*. (2024)
LSD							
Rodents	Adult male C57BL/6N mice	Unstimulated	30 μg/kg/day	• Kynurenine/tryptophan ratio, kynurenine/5-HT ratio, kynurenine levels • Endocannabinoid metabolites	↓ kynurenine levels, decrease in kynurenine/5-HT ratio • No effect on 5-HT levels • Modulation of endocannabinoid metabolites.	Kynurenine pathway	Inserra *et al*., (2023)
*N,N*-DMT							
Rodents	Male Wistar rats	Middle cerebral artery occlusion	1 mg/kg + 2 mg/kg/h intraperitoneally	• BDNF expression • Apoptotic cascade activation • Pro-inflammatory cytokine levels	↑ Expression of both mRNA and protein levels of APAF-1 ↑ BDNF expression ↓ TNF-α, IL-1β, Il-6 expression ↑ IL-10 expression	5-HT_2A_R signalling and Sigma1-R	Nardai *et al*. (2020)
	Male Sprague-Dawley rats	Cerebral forebrain ischaemia	1 mg/kg/h continuous infusion through left femoral vein	• Number of apoptotic cells • Astrocytic survival • Microglial activation	↓ Number of apoptotic cells • Rescue of astrocytes • Microglial activation was not modulated by *N,N*-DMT	Sigma1-R	Szabo *et al*. (2021)
	Male C57BL/6 mice	Amyloid-β induced AD	1 mg/kg intraperitoneally	Glial activation	• Alleviation of astrocytic activation and astrogliosis in the hippocampus and dentate gyrus • No attenuation of microglial activation.	5-HT_2A_R signalling and Sigma1-R	Borbély *et al*., (2022)
	Male Sprague-Dawley rats	Predator exposure and psychosocial stress-induced PTSD	2 mg/kg intraperitoneally	Pro-inflammatory cytokine mRNA expression and receptor expression	↓ Expression of IL-1β, IL1r1, TLR4, TLR6 and TLR7	5-HT_2A_R signalling and Sigma1-R	Kelley *et al*. (2022)
Humans	Adult male humans	Unstimulated	1 mg/kg	• Serum cortisol levels • Blood lymphocyte subpopulations	↑ Serum cortisol levels ↓ CD3 and CD4 cells percent ↑ NK cells percent • No changes in CD8 and CD19 levels.	HPA-axis modulation	dos Santos *et al*. (2011)
Ayahuasca							
Rodents	Male Wistar rats	Caecal ligation sepsis	[1–4] mL/kg	• Expression of inflammatory cytokines, BDNF, and oxidative stress markers • Sepsis-induced anxiety-like behaviour	↓ Expression of mRNA and protein levels of APAF-1 ↑ BDNF mRNA levels ↓ TNF-α, IL-1β, and IL-6 mRNA levels ↑ IL-10 mRNA levels ↓ Nitric oxide signalling ↓ Anxiety-like behaviour	5-HT_2A_R signalling	de Camargo *et al*. (2024)
Humans	TRD in adult male and female humans	Unstimulated	Ayahuasca 1 ml/kg *N,N*-DMT equiv. 0.36 mg/kg	• Salivary cortisol levels 48h after dosing • MADRS score	No change in MADRS score, cortisol levels similar to healthy controls after dosing Lower cortisol levels in patients before dosing when compared to control	HPA axis modulation	Galvao *et al*. (2018)
	TRD in adult male and female humans	Unstimulated	Ayahuasca 1 ml/kg *N,N*-DMT equiv. 0.36 mg/kg	• C-reactive protein levels • MADRS score • IL-6 plasma levels	• Reductions in C-reactive protein was correlated with lower depressive symptoms in both treatment- resistant depression participants and healthy control groups • No changes in IL-6.	• 5-HT_2A_R modulation and signalling and Sigma1-R. • HPA axis modulation	Galvao -Coelho et al., (2020)
5-MeO-DMT							
Humans	Adult male and female humans	Unstimulated	[17–61] mg inhalation	• Salivary IL-6 • Cortisol levels	↓ Salivary IL-6 ↑ Salivary cortisol	• Psycho-neuroimmune feedback effects 5-HT_2A_R and Sigma1-R, or • increased cortisol	Uthaug *et al*. (2020)
(*R*)-DOI							
Rodents	Male Wistar rats	Poly I:C	1 mg/kg intraperitoneally	Poly I:C induced fatigue	No effect	TLR3	Katafuchi *et al*. (2005)
	CBA mice	Single dose of sheep erythrocytes	1 mg/kg intraperitoneally	CD8+ T-cell count	↓ CD8+ T cells in spleen and periphery b • Blocked by ketanserin	5-HT_2A_R signalling	Davydova *et al*. (2010)
	BALB/c mice	TNF-α induced inflamed post-capillary venules	10 μM	Eosinophil intracellular Ca^2+^ levels	↑ Intracellular Ca^2+^	ROCK, MAPK, PI3K and the PKC-calmodulin pathway	Kang *et al*. (2013)
	Young adult male C57BL/6J mice	Intraperitoneal injection of TNF-α	[0.01–0.3] mg/kg intraperitoneally	Circulating IL-6 and VCAM-1 expression	↓ IL-6 and VCAM-1 expression	5-HT_2A_R signalling	Nau *et al*. (2013)
	BALB/c mice	Ovalbumin-induced chronic asthma	1 mg/kg nose-only inhalation	• Airway hyperresponsiveness • Pulmonary inflammation	↓ Pulmonary inflammation ↑ Airway hyperresponsiveness ↓ IL-15 and IL-9 ↑ IL-13 and IL-33	5-HT_2_R signalling	Flanagan *et al*. (2019a)
	Young adult male ApoE^−/−^ mice in a C57BL/6 genetic background	High-fat diet	0.15 μg/h subcutaneous infusion	• Pro-inflammatory gene expression • Glucose homeostasis • Circulating cholesterol	• Attenuated increase of HF-diet-induced IL-6, Cxcl10, and TNF-α. • Normalised glucose homeostasis ↓ Circulating cholesterol	5-HT_2A_R signalling	Flanagan *et al*. (2019b)
	Respiratory-pathogen-free Brown Norway rats	Ovalbumin-induced chronic asthma	[0.001–1] mg/kg nose-only inhalation or intraperitoneally	Pro-inflammatory gene expression in whole lung	↓ IL-5, IL-6, TNF-α, IL-1β	5-HT_2A_R signalling	Flanagan *et al*. (2021)
	Male adult C57BL/6 mice	Intraperitoneal injection of LPS	2 mg/kg intraperitoneally	• Dendritic spine density in PFC, and regions of the hippocampus. • Depressive-like behaviour • LPS-induced splenomegaly	• No effect on decreased dendritic spine density • No effect on immobility time in the FST • No effect on reversing LPS-induced splenomegaly	n/a	Qu *et al*. (2023)
	Male C57BL/6 J mice	Unstimulated	1 or 30 mg/kg/day	• Glial marker expression • Cytokine expression in glia	↑ Expression of IL-6, IBA1 and TNF-α	5-HT_2C_R signalling	Custodio *et al*. (2023)
	Male adult C57BL/6 mice	LPS or chronic restraint stress	2 or 4 mg/kg	• Depressive-like behaviour • Circulating IL-6 plasma levels • LPS-induced splenomegaly	• No effect on immobility time in the FST • No effect in the sucrose preference test • No effect seen on LPS-induced splenomegaly ↓ Circulating IL-6 plasma levels • No changes in LPS-induced PSD-95 protein reductions.	n/a	Liu *et al*. (2023)
	Male C57BL/6NHsd mice	Stress Alternatives Model	[0.015–0.3] mg/kg subcutaneously	• Cytokine gene expression in plasma and CSF • Anxiety-like behaviour	↓ Plasma and limbic brain levels of TNF-α ↓ freezing response	HPA axis modulation	Krupp *et al*. (2024)
	Pathogen-free wild-type BALB/c mice	Ovalbumin-induced chronic asthma	[0.1–3] mg/kg nebuliser	Pro-inflammatory gene expression in whole lung	Attenuated elevation of OVA-induced IL-6, Cxcl10, and Arg-1	5-HT_2A_R signalling	Flanagan *et al*. (2024)

## Translational application for disorders in humans

Research into the possible therapeutic use of psychedelics has surged in recent years, both in clinical and preclinical research. Landmark clinical trials have found that a single dose of psilocybin is sufficient for a rapid and long-lasting anti-depressant effect in patients with MDD and TRD, even with concomitant SSRI medication (Ross *et al*., 2016; Griffiths *et al*., 2016; Carhart-Harris *et al*., 2017, 2018, 2021; Goodwin *et al*., 2022, 2023b ; Raison *et al*., 2023). It was recently reported that the intensity of the psychedelic experience is correlated with depression response as measured by the Montgomery–Åsberg Depression Rating Scale (MADRS) (Goodwin *et al*., 2025).

Results reported in the largest phase II double-blind, parallel group, randomised clinical trial so far, suggest that previously reported antidepressant effects may have been inflated. The 25 mg dose of psilocybin had a 37% response rate three weeks after dosing according to the MADRS, compared to 18% with the 1 mg dose (Goodwin *et al*., 2022). Small sample sizes and other limitations commonly found in psychedelic research have led to larger and more robust phase III clinical trials that are underway at time of writing. However, results so far are promising for treatment of psychiatric illnesses.

Recent evidence suggests that psychedelics and psychedelic-like agents, including ketamine, may serve as novel interventions for patients who do not respond to conventional treatments (Kiraly *et al*., 2017; Quintanilla *et al*., 2024). Current research is exploring psilocybin’s potential to treat neurological conditions involving immune system imbalances, such as fibromyalgia and functional neurological disorder (Butler *et al*., 2024; Bornemann *et al*., 2024). The question remains as to which patients will see the most beneficial effects from psychedelics, and whether there may be biomarkers that could be used to predict responses from psychedelic-assisted therapy. As explored in this review, these biomarkers could include immune, hormonal, neuronal, or even microbiota parameters (Kelly *et al*., 2023). Future biomarker detection could use less invasive methods, such as wearable device technology for example.

Additionally, the optimal treatment regimen for psychedelic therapy remains undetermined. Although major clinical studies have used either one or two dosing sessions, the potential for periodic retreatment – perhaps every six to twelve months – for those who have previously gone through psychedelic-assisted therapy needs to be further investigated. In any case, psychedelic-assisted therapy will inherently be different from conventional antidepressant treatments that require daily consumption and often produce persistent side effects. Additionally, microdosing of psychedelics may offer similar beneficial effects without the acute subjective effects, although further double-blind research is required to validate this regimen (Kinderlehrer, 2025).

The immune system and the CNS maintain a dynamic interplay mediated through immune cells and organs, and the HPA axis (Bellavance and Rivest, 2014; Malek *et al*., 2015). Dysregulation of communication networks between these systems is implicated in a spectrum of mental health disorders, including depression (Arteaga-Henriquez *et al*., 2019; Lynall *et al*., 2020; Lamers *et al*., 2020; Zeng *et al*., 2024; Jarkas *et al*., 2024; Hagenberg *et al*., 2025; Penninx *et al*., 2025). A meta-analysis conducted by Osimo *et al*. (2019) revealed that approximately 25% of individuals diagnosed with depression exhibit signs of low-grade inflammation. In lower-middle-income countries, the prevalence of elevated plasma CRP could be higher, with 87% of participants with treatment-resistant depression displaying low-grade inflammation (Fellows *et al*., 2024). The authors suggest that targeting inflammatory symptoms individually may improve treatment outcomes in this cohort.

According to a recent meta-analysis, CRP was higher in females but not males with depression when compared to healthy controls; however, the sex effect did not reach significance (Jarkas *et al*., 2024). When disregarding sex, CRP levels were increased slightly when compared to controls, but again this result did not reach significance. However, a recent study revealed that the presence of elevated CRP levels does not necessarily correlate with increased depression severity, indicating a complex relationship between inflammation and depressive symptoms (Suneson *et al*., 2023).

Elevated inflammatory markers among patients with depression have been well-documented in the literature, with particular emphasis on cytokines IL-1β, IL-6, and TNF-α, which have been associated with exacerbated depressive symptoms (Dantzer *et al*., 2008; Suneson *et al*., 2023; Hassamal, 2023). Cytokines can influence the secretory activity of the HPA axis, further intensifying inflammatory responses (Turnbull and Rivier, 1999; Schiepers *et al*., 2005). In addition, current therapeutic treatments for depression have demonstrated anti-inflammatory effects, although findings have been inconsistent (Horikawa *et al*., 2010; Tynan *et al*., 2012; Wang *et al*., 2019; Strawbridge *et al*., 2023), and the number of failed treatment trials for MDD has been associated with levels of inflammatory markers (Haroon *et al*., 2018), hinting at an interaction between the immune system and antidepressant efficacy. Further work is required to understand the potential relevance of chronic low-level inflammation to the serotonergic system in a significant subset of the depressed population. Given the role of 5-HT in both the central nervous and immune systems, 5-HT_2A_R psychedelics present a potential avenue for modulating central and immune system responses.

Clinical trials have increasingly pointed to significant alterations in brain functional connectivity and the default mode network following psychedelic administration, as observed through magnetic resonance imaging (MRI) (Carhart-Harris *et al*., 2017; Mertens *et al*., 2020; Doss *et al*., 2021). Animal models and *in vitro* studies have also suggested enduring neuroplastic effects, including enhanced synaptogenesis (Ly *et al*., 2018; Raval *et al*., 2021; Moliner *et al*., 2023; Purple *et al*., 2024; Schmidt *et al*., 2024; Duque *et al*., 2024). In an LPS-induced mouse depressive-like model (*R*)-ketamine and the non-hallucinogenic LSD analogue lisuride led to an antidepressant-like effect in the forced-swim test, a commonly used preclinical test of antidepressant activity (Qu *et al*., 2023). Interestingly, (*R*)-DOI by itself had no effect on the LPS-induced depressive-like behaviour in this test. Both (*R*)-ketamine and lisuride prevented the LPS-induced decrease in dendritic spine density in the prelimbic area of the mPFC, the CA3 and the dentate gyrus regions of the hippocampus, suggesting a potential for preserving neuronal connectivity within the hippocampus.

Liu *et al*. (2023) assessed the effect of pre-treatment with (*R*)-ketamine, (*R*)-DOI, and lisuride, in two different models of depressive-like behaviour in mice, either using systemic LPS administration or chronic restraint stress (CRS). In both models, an increase in the immobility time in the FST, and a decrease in the sucrose preference test, were measured. Interestingly, (*R*)-ketamine, but not (*R*)-DOI or lisuride, had a significant antidepressant-like effect in both models. However, pre-administration of (*R*)-DOI, lisuride, and (*R*)-ketamine led to decreased circulating plasma IL-6 levels in the LPS model. In both models, (*R*)-ketamine was the only pre-treatment that blocked the reduction in postsynaptic density-95 (PSD-95) expression, a protein critical for synaptic plasticity.

Additionally, as 5-HT_2A_R is expressed in the hypothalamic paraventricular nucleus, psychedelics may directly trigger ACTH release, which in turn drives cortisol release from the adrenal glands (Zhang *et al*., 2002). This effect has been reported in preliminary studies in humans with increased ACTH and cortisol levels after psychedelic treatment, indicative of HPA axis activation (Hasler *et al*., 2004; Galvao *et al*., 2018; Galvao-Coelho *et al*., 2020; Uthaug *et al*., 2020). Modulation of the HPA axis may contribute to central and immune system responses to psychedelics (Schindler *et al*., 2018; Johnston *et al*., 2023). Recent results in healthy mice suggest that the psilocybin-induced short- and long-term anxiolytic effects result from psilocybin-induced glucocorticoid release (Jones *et al*., 2023). Indeed, the anxiolytic effect of psilocybin was blunted by pre-treatment with a glucocorticoid receptor antagonist. In mice, psilocybin was found to increase corticosterone production in both males and female mice (Farinha-Ferreira *et al*., 2025) producing anxiolytic-like effects. These results support the idea that the HPA axis may be directly involved with the anxiolytic effects observed with psychedelics.

Peripheral inflammation and chronic stress alter tryptophan metabolism via kynurenine pathway induction, leading to the production of neuroactive metabolites (Barone, 2019; Castro-Portuguez and Sutphin, 2020; Salminen *et al*., 2020; Brown *et al*., 2021), where the induction of IDO, the rate-limiting enzyme in the kynurenine pathway, plays a key role (O’Connor *et al*., 2009). However, there is little evidence to indicate that the 5-HT_2A_R influences the kynurenine pathway. Inserra *et al*. (2023) found that LSD decreased hippocampal levels of kynurenine and endocannabinoid related metabolites in the mouse brain. These results suggest that 5-HT_2A_R-mediated central effects may influence metabolites from the kynurenine pathway, and that psychedelics may modulate kynurenine metabolism. Further studies are required to determine the extent to which the kynurenine pathway is involved in the biological effects of psychedelics.

Rijsketic *et al*. (2023) examined the impact of environmental context on psilocybin-induced neural activity in mice by measuring immediate early gene expression. Both the environmental context and psilocybin elicited independent brain-wide neural responses, but there was little synergistic interaction between the two (Rijsketic *et al*., 2023).

Using (*R*)-DOI, a recent study was able to isolate and stimulate psychedelic-responsive neurons located in the mPFC of mice. Stimulating these mimicked the anxiolytic effects of (*R*)-DOI without inducing the HTR, providing evidence that the anxiolytic and hallucinogenic effects of psychedelics could be dissociated (Muir *et al*., 2024).

## Future directions

Neuroinflammation, along with neuronal atrophy and death, significantly contributes to the development of many disorders, including depression (Dantzer *et al*., 2008; Osimo *et al*., 2019; Suneson *et al*., 2023), stroke (Candelario-Jalil *et al*., 2022), neurodevelopmental disorders (Han *et al*., 2021), and neurodegenerative diseases (Teleanu *et al*., 2022; Singh, 2022). Additionally, the BBB is frequently compromised in various neurodegenerative conditions (Ruan *et al*., 2022; Sulimai *et al*., 2023; Hang *et al*., 2023; Bruno *et al*., 2024). A recent study found that patients with long-COVID-associated brain fog exhibited persistent systemic inflammation with BBB disruption (Greene *et al*., 2024). Additionally, it was reported that long-COVID was associated with 5-HT deficiency, possibly attributed to viral-induced inhibition of intestinal amino acid absorption (Wong *et al*., 2023). These studies indicate a direct link between a viral challenge on the immune system and its likely long-term effects on the CNS.

Measuring neuroinflammation, BBB permeability, and neuronal death is challenging in both animal models and patients. However, neuroimaging techniques, such as positron emission tomography (PET), MRI, and computed tomography (CT) scans, can be used in preclinical and clinical studies to assess these measures. Recent advancements in MRI modalities have enabled researchers to measure enlarged perivascular spaces, BBB permeability, cerebral perfusion, and neuroinflammation (Rowsthorn *et al*., 2023; Kim *et al*., 2023). Advances in PET now allow the measurement of tracers associated with neuroinflammation (Masdeu *et al*., 2022).

These imaging techniques can also be deployed to evaluate the effects of psychedelics. Although MRI scans have been utilised in clinical trials for psychedelics in depressed and healthy participants (Sanches *et al*., 2016; Carhart-Harris *et al*., 2017; Mertens *et al*., 2020; Doss *et al*., 2021; Shinozuka *et al*., 2024). A recent study even demonstrated, in a small sample size, that treatment response can be predicted using functional connectivity (Copa *et al*., 2024). However, neuroinflammation has not yet been assessed in this context.

A significant advantage of these imaging methods is that they can be applied similarly in animal models. MRI and other imaging modalities may provide imaging markers to assist in monitoring the long-term effects of psychedelics in animal models and patients. This approach can help ensure the safety and efficacy of these treatments in clinical practice and optimise therapeutic outcomes.

As previously highlighted, potency and affinity values vary greatly between 5-HT_2A_R psychedelics. There is a lack of pre-clinical and clinical studies that directly compare their effects on the immune and CNS. Exploring their possible differences could help researchers and clinicians understand the receptors and cellular pathways involved in the immunomodulatory and antidepressant properties of these compounds. Additionally, studies discussed in this review draw attention to the lack of direct comparison between unstimulated and stimulated immune systems, whether in isolated cells or whole biological systems. This type of study would be valuable understanding how psychedelics are immunoregulatory, and not simply anti- or pro-inflammatory.

## Conclusion

*In vitro* and *in vivo* studies have investigated the effects of 5-HT2_A_R psychedelics at supraphysiological concentrations and have shown immune-modulating properties of these compounds. Receptors beyond the serotonergic family, such as the TrkB receptor, may play a key role in mediating the immunomodulatory effects. However, well-powered clinical studies are lacking, making it challenging to evaluate the immunomodulatory properties of psychedelics in humans – both in healthy individuals and those with psychiatric disorders. Further research is necessary to elucidate these effects and their connection to therapeutic outcomes. MRI and other brain imaging techniques offer a valuable translational tool to bridge the gap between preclinical and clinical studies and to advance our understanding of the effects of psychedelics in the brain.
